# Genome-Wide Analysis of the *KNOX* Gene Family in *Malus sieversii*

**DOI:** 10.3390/plants15081152

**Published:** 2026-04-09

**Authors:** Da Zhang, Guodong Zhao, Dongmei Chen, Tongsheng Zhao, Linguang Jia, Han Wang, Xin Liu, Bowei Zhu, Gang Niu, Xinsheng Zhang, Chao Zhao, Chaohong Zhang

**Affiliations:** 1 Changli Institute of Pomology, Hebei Academy of Agriculture and Forestry Sciences, Qinhuangdao 066600, China; d.zhang@nwafu.edu.cn (D.Z.); guodong19823@163.com (G.Z.); chendm2009@126.com (D.C.); tshzh71@163.com (T.Z.); dsjialinguang2020@163.com (L.J.); 15830298656@163.com (H.W.); 18830180711@163.com (X.L.); 18331561549@163.com (B.Z.); niug@nwafu.edu.cn (G.N.); 2 Shijiazhuang Institute of Pomology, Hebei Academy of Agriculture and Forestry Sciences, Shijiazhuang 050000, China

**Keywords:** *Malus sieversii*, *KNOX*, gene family, anthocyanin

## Abstract

*KNOTTED1-LIKE HOMEOBOX* (*KNOX*) genes are conserved transcription factors that play crucial roles in plant growth, development, and stress responses. However, systematic characterization of the *KNOX* family in *Malus sieversii*, a valuable germplasm resource with outstanding stress tolerance and flavonoid accumulation, remains lacking. In this study, we performed a genome-wide identification of the *KNOX* gene family in *M. sieversii* and identified 21 *MsiKNOX* genes. Phylogenetic analysis classified these genes into three subfamilies (Class I, II, and M), with structural features and motif compositions consistent with those of their orthologs in *Arabidopsis thaliana* and cultivated apple. Chromosomal localization revealed an uneven distribution across 13 chromosomes, and synteny analysis indicated both conserved evolution and lineage-specific expansion of the *KNOX* family in *M. sieversii*. Promoter cis-element analysis suggested that *MsiKNOX* genes are potentially involved in responses to multiple abiotic stresses and hormone signaling. Expression profiling under ABA and GA treatments showed that most *MsiKNOX* genes responded differentially to these phytohormones. Notably, *MsiKNOX09* was significantly upregulated by ABA and downregulated by GA, and was further shown to physically interact with the anthocyanin-associated MsiMYB1 in yeast two-hybrid and split-luciferase assays. These findings provide a comprehensive overview of the KNOX gene family in *M. sieversii* and suggest that *MsiKNOX09* acts as a hormone-responsive regulator and may participate in *MsiMYB1*-mediated regulatory pathways.

## 1. Introduction

Apple production faces serious challenges from inbreeding depression. The narrowing of the genetic base has led to cultivated apples with poor nutritional quality and low flavonoid content. Recently, there has been growing recognition of the critical role of *Malus sieversii* in apple domestication and breeding [1,2]. *M. sieversii*, commonly referred to as “Xinjiang wild apple”, is distributed mainly in the Tianshan Mountains across China, Kazakhstan, Kyrgyzstan, and Uzbekistan [3,4]. As the wild ancestor of cultivated apple, it represents an elite genetic resource for improving both stress resistance and flavonoid-related traits in apple breeding [1,5]. Therefore, identifying key genes regulating flavonoid accumulation in *M. sieversii* is of great importance for its conservation and utilization.

*KNOTTED1-LIKE HOMEOBOX* (*KNOX*) genes, members of the three amino acid loop extension (TALE) homeodomain superfamily, are typically characterized by four conserved domains—KNOXI, KNOXII, ELK, and HD—although the ELK and HD domains are absent in some cases [6]. KNOX is a conserved transcription factor (TF) family that is widely distributed among sequenced plants, including *Arabidopsis thaliana*, *Malus domestica*, *Oryza sativa*, *Solanum lycopersicum*, *Populus trichocarpa*, and *Zea mays* [7,8,9,10]. Based on sequence similarity, phylogenetic analysis, expression patterns, and intron position, *KNOXs* can be categorized into two subclasses: class I and class II [11]. Additionally, *KNOX* lacking the HD domain was identified in *A. thaliana*, which defined a novel class designated KNATM [12]. In *A. thaliana*, four class I *KNOXs* (*SHOOT-MERISTEMLESS* [*STM*], *KNAT1*, *KNAT2*, and *KNAT6*) and four class II members (*KNAT3*, *KNAT4*, *KNAT5*, and *KNAT7*) have been identified. Class I *KNOXs* are well characterized for their roles in meristem maintenance, leaf blade morphogenesis, internode elongation, hormone homeostasis, and inflorescence patterning [13,14,15]. *KNAT1* is expressed in shoot and inflorescence tissues but not in leaves, while *KNAT2* shows high transcript levels in these tissues yet low abundance in leaves, affecting leaf morphology. *KNAT6*, by contrast, is expressed at lateral root initiation sites and plays a role in meristem activity and organ separation [16,17]. *STM* loss-of-function mutations lead to embryos devoid of a shoot apical meristem [18]. Class II *KNOXs* exhibit relatively pervasive expression patterns across diverse tissues and organs and primarily function in the regulation of plant organ differentiation and secondary cell wall biosynthesis [6]. *KNAT3*, *KNAT4*, and *KNAT5* function redundantly in root development and lateral organ differentiation [19]. Both KNAT3 and KNAT7 regulate mucilage biosynthesis in Arabidopsis seeds, thereby contributing to the development and properties of this essential seed component [20]. KNATM gene is specifically expressed in the proximal–lateral domains of organ primordia and along the edges of mature organs, playing a role in modulating leaf polarity and leaf traits [12,21].

*KNOXs* also play crucial roles in modulating plant metabolism, hormone signaling, and stress responses in crops [22,23,24]. KNOXs are involved in regulating target gene expression and interacting with other proteins [25,26,27]. MdKNOX19 enhances ABA sensitivity in apple calli through direct activation of *MdABI5* expression in *M. domestica* [23]. MdKNOX15 controls flowering time and plant height by modulating GA levels via activation of *MdGA2ox7* expression [28]. OsHOS59 negatively regulates plant architecture as well as rice grain size and glume cell length in *O. sativa* [29]. The DELLA protein SLR1 physically interacts with KNOX protein OsOSH1 to suppress OSH1-mediated activation of downstream genes involved in panicle development [30]. The BELL-KNOX module qSH1-OSH71 controls rice seed shattering by directly activating the xyloglucanase gene *OsXTH12* [31]. *KNOXs* coordinate spatiotemporal ripening in *S. lycopersicum* and shape a gradient of fruit chloroplast development through regulation of *GOLDEN2-LIKE* expression [9,32]. KNAT2/6b suppresses xylem differentiation through regulation of NAC TFs in *P. trichocarpa* [33]. KNOTTED1 activates the GA catabolism gene *GA2ox1* to establish a boundary between shoot meristem cells and rapidly elongating cells in *Z. mays* [34].

*M. sieversii* is widely appreciated for its outstanding adaptability to diverse environments and its rich flavonoid accumulation. While the KNOX family has been identified in *Malus domestica* [35], its characterization in *M. sieversii* remains undocumented. This study aims to identify and characterize the *KNOX* gene family in *M. sieversii* at the genome-wide level. Based on the high-quality apple genome data [36] and the *KNOX* classification system established in *A. thaliana*, a total of 21 *KNOX* genes were identified. Their gene structures, conserved motifs, subcellular localization, chromosomal distributions, phylogenetic relationships, and promoter cis-elements were systematically analyzed. Through RT-qPCR and protein interaction analysis, *MsiKNOX09* was preliminarily characterized. These findings provide a foundation for further functional studies and offer new insights for apple breeding.

## 2. Result

### 2.1. Identification and Phylogenetic Analysis of Members of the KNOX Gene Family in M. sieversii

By combining BLASTP and HMMER searches followed by manual curation, a total of 21 KNOX proteins were identified from the *M. sieversii* genome. Multiple sequence alignment of these MsiKNOX proteins revealed four conserved domains—KNOX1, KNOX2, ELK, and Homeobox_KN—which are typical of the plant KNOX family. Among these, the KNOX1 domain exhibited higher sequence conservation compared to the others (Supplementary Figure S1).

Using the ProtParam tool, we analyzed the physicochemical properties of the proteins encoded by these 21 genes, revealing significant differences among various KNOX protein sequences (Supplementary Table S1). The amino acid length ranged from 139 to 574 aa, with most sequences falling between 300 and 400 aa. Protein molecular weight ranged from 15.51 to 64.61 kDa. Isoelectric points varied from 4.67 to 6.67, with all proteins having an isoelectric point below 7, indicating that they are acidic to neutral proteins. This is consistent with their role as TFs, which typically require an acidic nature to interact with negatively charged DNA molecules [37]. The average hydrophilicity of the proteins ranged from −1.017 to −0.404, indicating that all KNOX family members are hydrophilic proteins. Subcellular localization predictions showed that all 21 members were predicted to localize in the nucleus, which aligns well with their core biological function as TFs—binding to chromosomal DNA and regulating downstream gene transcription. Notably, eight members (MsiKNOX06, 07, 08, 09, 13, 16, 17, and 18) were predicted to localize in both the cytoplasm and the nucleus. Additionally, MsiKNOX06 was further predicted to possibly localize in chloroplasts. These dual- or multi-localization predictions suggest that some KNOX proteins may undergo post-translational modifications, protein–protein interactions, or signal-mediated regulation, enabling dynamic shuttling between the cytoplasm and the nucleus to fine-tune their transcriptional activity.

**Figure 1:**
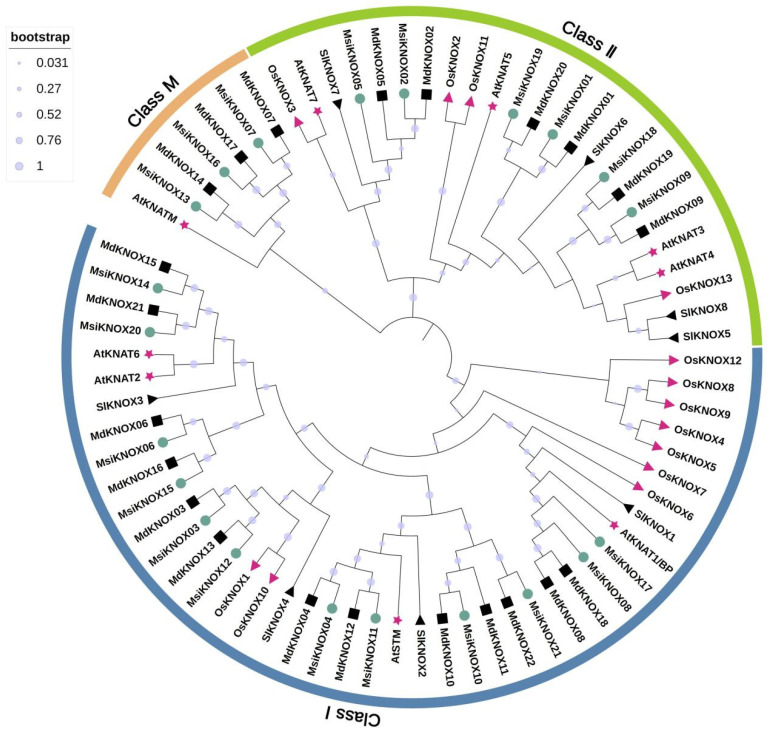
Systematic evolutionary analysis of KNOX family proteins. Neighbor-joining tree representing phylogenetic relationships among *KNOX* genes from A. thaliana, *M. sieversii*, *M. domestica*, *O. sativa*, and *S. lycopersicum.*

Additionally, a phylogenetic tree was constructed based on the full-length amino acid sequences of KNOX proteins from 22 *M. domestica* var. ‘Golden Delicious’, 9 *A. thaliana*, 13 *O. sativa*, 8 *S. lycopersicum*, and the 21 *M. sieversii* proteins identified in this study (Figure 1). Phylogenetic analysis classified the 21 *M. sieversii* KNOX proteins into three subfamilies. The Class I subfamily contained 12 members orthologous to functionally characterized *A. thaliana* genes such as *STM*, *KNAT1*, *KNAT2*, and *KNAT6*, and all possessed the typical KNOX1, KNOX2, ELK, and Homeobox_KN domains. The Class II subfamily consisted of six proteins, each containing the KNOX1, ELK, and Homeobox_KN domains. The Class M subfamily included three proteins orthologous to *AtKNATM*, which contained only the KNOX1 and KNOX2 domains. In *M. domestica*, 13 Class I, 6 Class II, and 3 Class M KNOX proteins were identified, suggesting a slight expansion or functional diversification of the Class I subfamily in *M. domestica*. Overall, the core KNOX family structure is conserved between *M. sieversii* and *M. domestica*.

### 2.2. Analysis of MsiKNOX Gene Structure, Motifs, and Domains

**Figure 2:**
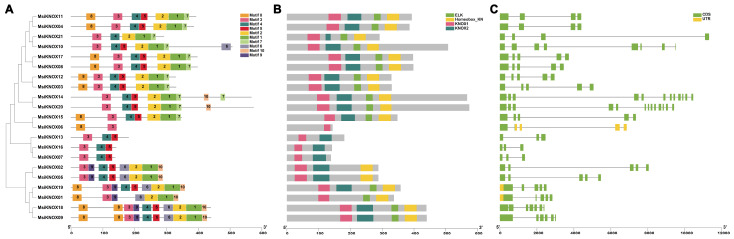
Schematic representation of protein and gene structures of *MsiKNOXs*. (**A**) Motifs 1–10 identified using the MEME search tool are marked on the protein sequences. (**B**) Analysis of conserved domains. (**C**) Analysis of the coding sequence and untranslated regions.

We conducted a gene structure analysis of *KNOX* genes in *M. sieversii* and found that most members exhibited considerable variation, containing 3 to 13 exons and 2 to 12 introns, with the majority possessing 5 exons and 4 introns. Additionally, only *MsiKNOX01*/*06*/*19* contained UTR sequences (Figure 2C).

Subsequently, we analyzed the conserved motifs of the KNOX proteins in *M. sieversii* and identified 10 conserved motifs, designated Motif 1 to Motif 10, ranging from 15 to 50 amino acids in length. These motifs were annotated using the Pfam and SMART databases, revealing that Motif 1, Motif 3, and Motif 4 correspond to the conserved domains of the Homeobox_KN, KNOX1, and KNOX2 superfamilies, respectively (Supplementary Figure S2). As shown in Figure 2A, all KNOX proteins in *M. sieversii* contain 2 to 10 conserved motifs. Among them, MsiKNOX09 and MsiKNOX18 possess the highest number of conserved motifs (10 each), whereas MsiKNOX06 contains the fewest (only Motif 3 and Motif 8). Phylogenetic analysis indicated that members with close evolutionary relationships generally share similar motif compositions. It was also observed that most KNOX proteins in *M. sieversii* contain the Motif 3, Motif 4, and Motif 5. Overall, the KNOX proteins in *M. sieversii* exhibit high conservation. The analysis of the conserved domains of the *M. sieversii* KNOX proteins is shown in Figure 2B, where all KNOX proteins contain a conserved KNOX1 domain; among them, MsiKNOX06 contains the only domain KNOX1.

### 2.3. Chromosome Mapping of MsiKNOXs

The chromosomal locations of the *MsiKNOXs* were obtained from the apple genome database, and a chromosome distribution map was generated using TBtools (v2.056, Supplementary Figure S3). The 21 *MsiKNOXs* were found to be unevenly distributed across 13 chromosomes. Specifically, chromosomes 2 (*MsiKNOX01*), 5 (*MsiKNOX04*), 9 (*MsiKNOX10*), 10 (*MsiKNOX11*), 12 (*MsiKNOX12*), 16 (*MsiKNOX20*), and 17 (*MsiKNOX21*) each contained a single gene. Chromosome 4 harbored two genes (*MsiKNOX02* and *MsiKNOX03*), and chromosome 15 harbored three genes (*MsiKNOX17*, *MsiKNOX18*, and *MsiKNOX19*). In summary, the apple *MsiKNOX* gene family shows a single-copy distribution in the genome.

### 2.4. Analysis of Cis-Acting Elements

**Figure 3:**
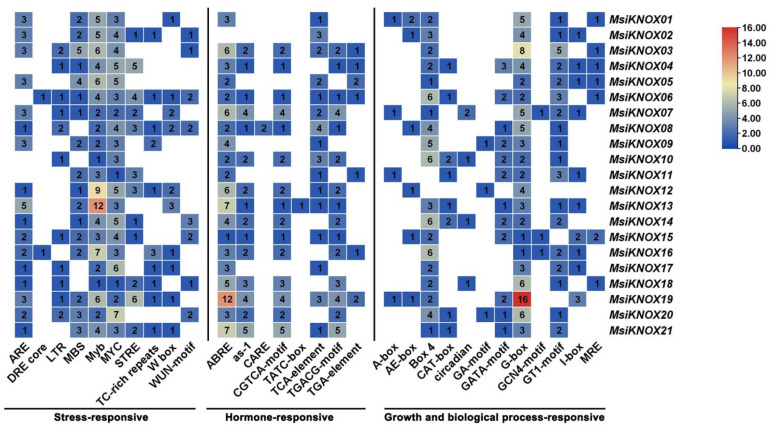
*Cis*-acting elements in the promoter regions of *MsiKNOXs*. The types of elements are displayed at the bottom, and the numbers indicate the quantity of each type.

Promoter analysis of the 2 kb upstream regions of the *KNOX* genes in *M. sieversii* was performed using PlantCARE (Figure 3). The results revealed that the promoter regions of the 21 *MsiKNOXs* contain 30 distinct types of cis-acting elements, including those involved in stress responsiveness, hormone signaling, and growth/development-related processes. The stress-responsive elements identified include Myb, MYC, ARE, LTR, and STRE; hormone-responsive elements include ABRE, as-1, and TGACG-motif; and growth/development-related elements include G-Box, Box 4, and GT1-motif. Among these, elements associated with hormone signaling and light responsiveness were more abundantly represented across the *MsiKNOX* promoters. Notably, *MsiKNOX13* harbors 12 MYB elements, while *MsiKNOX19* contains 12 ABRE elements and 16 G-box elements. These cis-element variations suggest that *MsiKNOXs* may play diverse roles in hormonal and environmental signaling pathways.

### 2.5. Collinearity Analysis of MsiKNOXs

**Figure 4:**
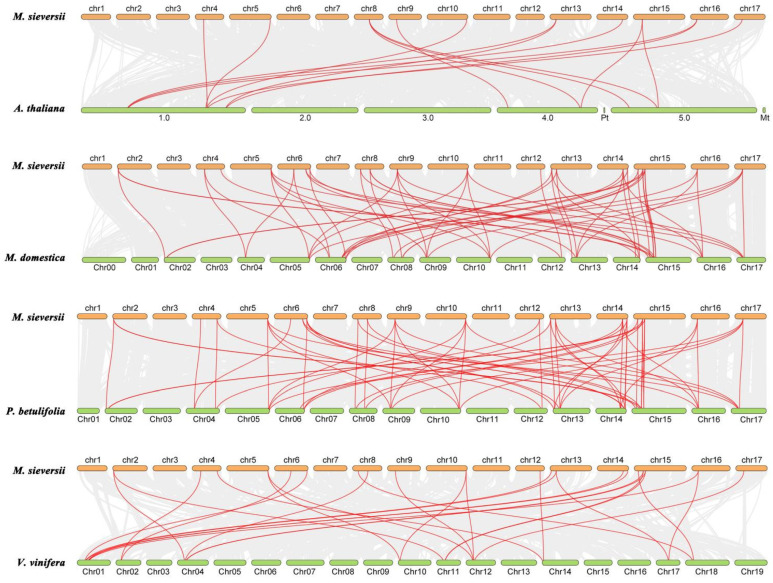
Collinear analysis of *M. sieversii*, *A. thaliana*, *M. domestica*, *P. betulifolia*, and *V. vinifera KNOX* family genes. Red lines indicate syntenic gene pairs.

To investigate the evolutionary dynamics of the KNOX gene family in *M. sieversii*, we performed comparative synteny analysis with four representative species: *A. thaliana*, *M. domestica*, *Pyrus betulifolia*, and *Vítis vinifera* (Figure 4). Additionally, *M. sieversii*, *V. vinifera*, and *P. betulifolia* were selected for collinearity analysis to reveal the evolutionary conservation and divergence of KNOX genes by integrating closely related Rosaceae species and a representative basal eudicot with an ancestral genome. The number of syntenic gene pairs varied considerably across species. The fewest pairs were detected between *M. sieversii* and *A. thaliana* (15 pairs), whereas the highest number was observed with *P. betulifolia* (62 pairs), suggesting a closer evolutionary relationship between the KNOX families of *M. sieversii* and *P. betulifolia*. A similar number of syntenic pairs was found with *M. domestica* (59 pairs), indicating conserved synteny within the Malus lineage. In contrast, only 27 syntenic pairs were identified with *V. vinifera*, comparable to the number observed with *A. thaliana*. When compared to the *A. thaliana* genome, 10 out of the 21 *MsiKNOXs* exhibited syntenic relationships, while the remaining 11 genes (*MsiKNOX01*/*03*/*05*/*06*/*07*/*08*/*12*/*13*/*16*/*17*/*19*) showed no detectable synteny. In comparison with *M.domestica*, all *MsiKNOX* genes except *MsiKNOX03* and *MsiKNOX12* displayed evidence of gene expansion, suggesting that most members of this family have undergone duplication events during the evolution of cultivated apple. Overall, these results indicate that the *KNOX* gene family has experienced both conserved evolution and lineage-specific expansion, with functional conservation maintained within *M. sieversii*.

### 2.6. Expression Analysis of Six MsiKNOXs

**Figure 5:**
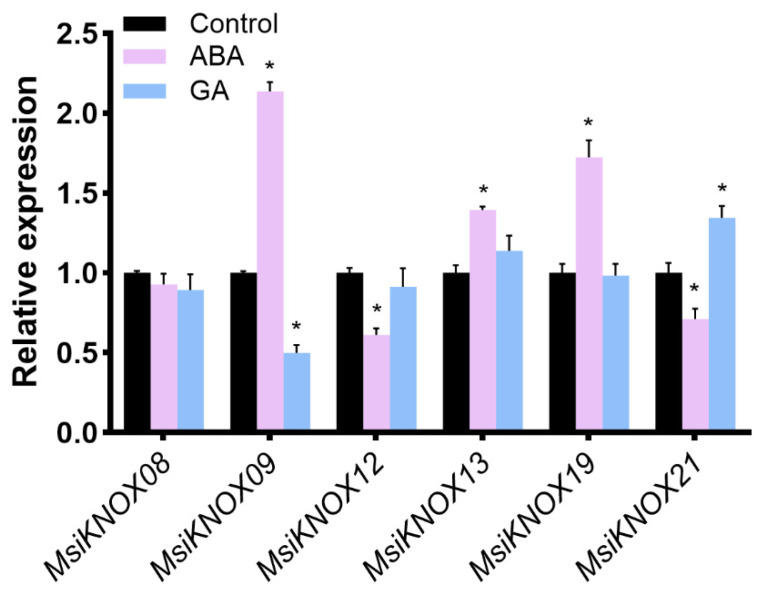
Quantitative expression levels of six *MsiKNOXs* under ABA and GA treatments. The x-axis represents different genes, and the y-axis shows the relative expression levels under various treatments, with the control value normalized to 1. One-way analysis of variance (ANOVA) followed by post hoc tests to determine significant differences between groups. All data are presented as mean ± standard deviation (SD) from at least three independent biological replicates. *, *p* < 0.05.

The genes were selected according to phylogeny, conserved motifs, gene structure, and expression levels. Only genes with complete conserved domains and distinct expression patterns were chosen for further analysis. Abscisic acid (ABA) and gibberellins (GA) are well-documented key regulators that antagonistically or synergistically control anthocyanin biosynthesis in various plant species [38]. To further investigate the response of *MsiKNOXs* to these hormones, we examined the expression patterns of six representative *MsiKNOXs* in *M. sieversii* under ABA and GA treatments (Figure 5). Under ABA treatment, the expression levels of *MsiKNOX09*, *MsiKNOX13*, and *MsiKNOX19* were significantly upregulated, whereas those of *MsiKNOX12* and *MsiKNOX21* were downregulated. Under GA treatment, *MsiKNOX09* was downregulated, while *MsiKNOX21* was upregulated compared to the control. *MsiKNOX08* expression remained unchanged under both treatments. Among these genes, *MsiKNOX09* exhibited the most pronounced response, with a fold change greater than two under both ABA and GA treatments. In conclusion, expression analysis of the six selected *MsiKNOXs* revealed that they respond differentially to ABA and GA treatments, suggesting their potential involvement in phytohormone-mediated regulatory networks.

### 2.7. Physical Interaction Between MsiKNOX09 and MsiMYB1

**Figure 6:**
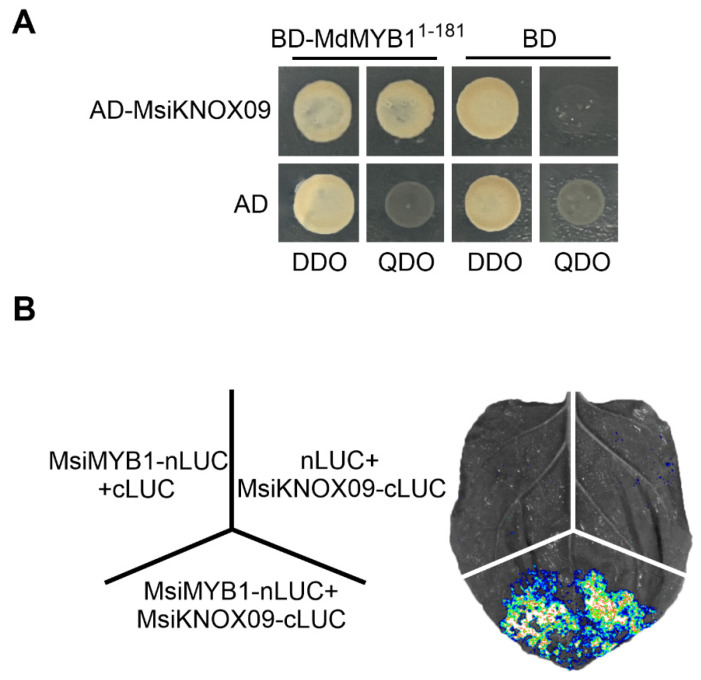
The validation of the protein interaction between MsiKNOX09 and MsiMYB1. (**A**). Yeast two-hybrid assay. (**B**). Luciferase assay. QDO and DDO refer to yeast deficient-type medium; DTO, SD/-Trp/-Leu. QDO, SD/-Trp/-Leu/-His/-Ade. The empty AD, BD, nLUC, and cLUC constructs serve as negative controls.

To preliminarily investigate whether MsiKNOX09 participates in the MsiMYB1-mediated signaling, we performed yeast two-hybrid (Y2H) and split-luciferase complementation assays. MsiMYB1 was chosen as a candidate interactor of MsiKNOX09 because it functions as a key MYB transcription factor that directly regulates anthocyanin accumulation and stress adaptation in apple and related species [38]. The coding sequences of *MsiMYB1* and *MsiKNOX09* were cloned into pGBKT7 and pGADT7, respectively. Co-transformation of these constructs into yeast strain Y2H Gold resulted in positive growth on SD/−Leu/−Trp/−His/−Ade medium, indicating a physical interaction between MsiMYB1 and MsiKNOX09 (Figure 6). This interaction was further confirmed by split-luciferase assays in *Nicotiana benthamiana* leaves, where co-infiltration of nLUC-MsiMYB1 and cLUC-MsiKNOX09 produced strong luciferase signals. Together, these results demonstrate that MsiMYB1 physically interacts with MsiKNOX09 in vitro and in vivo.

## 3. Discussion

*M*. *sieversii* is a tertiary relict fruit tree and the direct ancestor of cultivated apple (*M*. *domestica*), with extremely unique biological characteristics and important research value. It has undergone long-term natural selection in the Tianshan Mountains of Xinjiang, retaining rich genetic and phenotypic diversity that is significantly higher than that of modern cultivars. It possesses strong tolerance to drought, cold, poor soil and diseases, making it a core wild germplasm for apple stress resistance breeding. Furthermore, it shows unique morphological, reproductive and adaptive features distinct from cultivated apples [1,2]. As a unique and conserved TF family, KNOX was first identified in *Z. mays* and is highly conserved across land plants [35]. KNOX proteins play key roles in transcriptional regulation in land plants and are primarily localized in the nucleus [22]. Certain conserved TF families, such as bHLH and NAC, play essential roles in plant metabolism and stress tolerance during plant evolution, including the MYB and NF-Y gene families [39,40]. However, some TF families crucial for plant evolution remain poorly characterized. A deeper understanding of the transcriptional mechanisms and functions of KNOX genes is therefore needed. In this study, 21 *MsiKNOX* genes were identified in *M. sieversii*, one fewer than that in the cultivated apple cultivar ‘Golden Delicious’ (22 KNOX genes). This difference in gene number may reflect functional divergence shaped by distinct growth environments between the two species. *MsiKNOX* genes are predominantly distributed at the distal ends of chromosomes. This non-random localization pattern implies a potential correlation with their conserved roles in phytohormone signaling and stress adaptation, which may facilitate the rapid response of these genes to environmental cues (Supplementary Figure S3).

In this study, our phylogenetic analysis based on five representative land plant species suggests that KNOX genes have diverged into three distinct subfamilies early during land plant evolution (Figure 1). Researchers further classified KNOXs into three types based on the sequence similarity: phylogenetic analysis, expression patterns, and intron position. This classification is also applicable to wild *M. sieversii* and is consistent with the previous classifications in other land plants [13]. Gene duplication can arise from segmental duplication, random duplication, or retroposition [41]. Polyploidization in plants has resulted in the preservation of extensive duplicated chromosomal regions, among which segmental duplication is the most common mechanism driving gene family expansion [42]. Synteny analysis suggested that some KNOX genes in *M. sieversii* have been lost or expanded over evolutionary time (Figure 4).

Conserved motif analysis revealed that all 21 MsiKNOX proteins contain Motif 3. Structural analysis further confirmed that this motif resides within the conserved domain characteristic of the *KNOX* family, indicating that Motif 3 is a highly conserved element critical for KNOX protein functions (Figure 2). The *KNOX* genes in *M. sieversii* exhibit relatively complex structures, with only three members containing fewer than five exons—a feature highly similar to that observed in species such as *Toona fargesii* [43] and *Ipomoea batatas* [44]. Genes containing multiple introns have the potential to generate multiple protein variants through alternative splicing, which may contribute to functional diversification and evolutionary flexibility. Future studies using transcriptome data from *M. domestica* or *M. sieversii* will be needed to identify and validate potential splice forms of *KNOX* genes.

Cis-acting elements serve as TF binding sites that enable plants to adapt to environmental stress by modulating transcriptional activity. In *M. sieversii*, 11 *MsiKNOX* genes contain low-temperature responsive elements, and all 21 *MsiKNOXs* harbor MYB TF binding sites associated with drought induction. Additionally, multiple hormone-responsive cis-elements (e.g., ABRE, auxin, and jasmonic acid motifs) were identified in their promoters (Figure 3). These findings suggest that *MsiKNOXs* may not only actively respond to diverse abiotic stresses but also enhance stress tolerance by modulating the synthesis of related phytohormones. *M. domestica* is taxonomically closely related to *M. sieversii*, and its genome has been well assembled and annotated, which provides a high-quality and reliable reference for sequence alignment, gene identification and evolutionary analysis in this study [36]. The higher number of syntenic pairs between *M. sieversii* and *P. betulifolia* than between *M. sieversii* and *M. domestica* may be due to intensive artificial selection, chromosomal rearrangements, and gene loss during apple domestication, which have altered the genome structure of cultivated apples. By contrast, *P. betulifolia* maintains a relatively ancestral genome, showing stronger synteny with the wild species *M. sieversii*.

It has been shown that the regulation of stress responses is intimately connected with anthocyanin accumulation. In *A. thaliana*, AtMYB111 modulates salt responses by regulating anthocyanin accumulation [39]. AtMYB112 promotes anthocyanin formation during salinity and high light stress [45]. In *Prunus avium*, PacMYBA promotes ABA-mediated anthocyanin accumulation [46]. Furthermore, anthocyanin accumulation induced by diverse external stimuli is often associated with MYB1-related regulatory pathways in *M. domestica*. MdMYB308L promotes cold tolerance and anthocyanin accumulation [47]. MdWRKY40 interacts with MdMYB1 to promote wounding-mediated anthocyanin accumulation [48]. MdbZIP44 promotes ABA-mediated anthocyanin accumulation through the interaction with MdMYB1 and enhances the binding of MdMYB1 to the downstream targets [49]. MdERF38 promotes anthocyanin accumulation in response to drought stress by facilitating the binding of MdMYB1 to its target genes [50]. In this study, MsiKNOX09 interacts with MsiMYB1 protein (Figure 6). Expression analysis of six selected *KNOX* genes revealed that most responded to the treatments applied. Notably, *MsiKNOX09* was significantly induced by ABA but repressed by GA (Figure 5). Accordingly, these results imply that *MsiKNOX09* may be involved in drought or salinity stress responses in *M. sieversii*, possibly by regulating MsiMYB1-mediated signaling, which provides a clue for further investigating its function as a potential regulatory transcription factor, for which subcellular localization and transcriptional activity assays are essential. To further clarify the biological functions of *MsiKNOX09*, functional verification experiments such as overexpression and CRISPR/Cas9-mediated gene editing will be conducted in subsequent research. Notably, the absence of specific conserved domains typically present in KNOX family members may be associated with functional divergence or specialization; further experimental evidence is needed to clarify their biological significance.

## 4. Material and Methods

### 4.1. Identification of KNOX Gene Family Members in M. sieversii

To identify *KNOX* family members in *M. sieversii*, a BLASTP search was performed using *A*. *thaliana* KNOX protein sequences as queries against the *M. sieversii* proteome, implemented in TBtools (v2.056) [51]. Redundant sequences were removed, and candidate proteins were aligned using DNAMAN to verify uniqueness. Conserved domains were initially assessed using the NCBI CDD (https://www.ncbi.nlm.nih.gov/cdd, accessed on 1 April 2026). Proteins lacking the RGRP conserved domain were discarded. The remaining candidates were further validated using PFAM (http://pfam.xfam.org/, accessed on 1 April 2026) and SMART (https://smart.embl.de/, accessed on 1 April 2026) to confirm the presence of characteristic KNOX domains, ultimately defining the complete set of *KNOX* genes in *M. sieversii*. The genomic information of *M*. *sieversii*, *A. thaliana*, *M. domestica*, *P. betulifolia*, *V. vinifera*, *O. sativa*, and *S. lycopersicum* is listed in Supplementary Table S2.

### 4.2. Phylogenetic Analysis of the KNOX in M. sieversii

Multiple sequence alignment was performed using ClustalW (v2.0) integrated in MEGA X (v10.2.4) [52]. A phylogenetic tree was constructed via the Neighbor-Joining (NJ) method with *M. sieversii* and *A. thaliana* KNOX family members. The bootstrap test was conducted with 1000 replicates to assess node reliability.

### 4.3. Chromosomal Localization of MsiKNOXs

Based on the retrieved genomic information, the chromosomal locations of all KNOX motif genes in *M. sieversii* were determined, and a chromosomal localization map was generated.

### 4.4. Analysis of Gene Structure, Conserved Motifs and Phylogenetic of MsiKNOXs

Gene structures of the *M. sieversii KNOX* family were analyzed using the GSDS 2.0 online tool (http://gsds.cbi.pku.edu.cn/, accessed on 1 April 2026). Conserved motifs of KNOX proteins were identified with the MEME suite (https://meme-suite.org/meme/, accessed on 1 April 2026). To obtain *M. sieversii KNOX* gene sequences, a homology search was conducted by aligning *A. thaliana* KNOX protein sequences against the *M. sieversii* CDS database. Based on the combined *KNOX* gene sequences from both species, an evolutionary tree was constructed using MEGA X and visualized with the iTOL online tool (https://itol.embl.de/, accessed on 1 April 2026). The tree was built using the Maximum Likelihood method with 1000 bootstrap replicates.

### 4.5. Cis Acting Element Analysis

Promoter sequences (2000 bp upstream of the start codon) of *KNOX* family genes were extracted from the *M. sieversii* genome database. Cis-acting regulatory elements were predicted using PlantCARE (http://bioinformatics.psb.ugent.be/webtools/plantcare/html, accessed on 1 April 2026). Data analysis was performed with Excel 16.0, and the identified cis-elements were visualized using TBtools (v2.056) [51].

### 4.6. Real Time Fluorescence Quantitative (RT-qPCR) Analysis

ABA solution at a concentration of 100 μM and gibberellic acid GA_3_ solution at a concentration of 100 μM were prepared using distilled water containing 0.05% (*v*/*v*) Tween-80. Fourteen-day-old uniformly grown apple seedlings were treated with GA3 and ABA, respectively. *M. sieversii* seedlings were grown in tissue culture rooms at 25 ± 2 °C under a 16 h light/8 h dark photoperiod with a light intensity of 100–200 μmol m^−2^ s^−1^. Samples were immediately frozen in liquid nitrogen and stored at −80 °C for subsequent analysis. Gene-specific primers were designed using Primer3.0 software and synthesized by Sangon Biotech (Shanghai, China). Actin was used as the internal reference gene. Total RNA was extracted using the RNA plant Kit (Transgene, Beijing, China), and cDNA was synthesized with the PrimeScript RT reagent Kit (Takara, Shiga, Japan) following the manufacturer’s instructions. Each experiment was performed with three biological replicates. Relative gene expression levels were calculated using the 2^−ΔΔCT^ method. All RT-qPCR primers are listed in Supplementary Table S3.

### 4.7. Yeast Two-Hybrid Assay

The coding sequences of *MsiKNOX09* and *MsiMYB1* were amplified from *M. sieversii* cDNA and cloned into pGADT7 and pGBKT7 vectors, respectively, to generate fusion expression constructs. The resulting plasmids (BD-MsiMYB1 and AD-MsiKNOX09), along with empty BD and AD vectors as controls, were co-transformed into yeast competent cells. Transformants were first plated onto SD/−Trp/−Leu double-dropout medium and incubated at 30 °C for 4–5 days. Six individual colonies from each transformation were then streaked onto SD/−Trp/−Leu/−His/−Ade quadruple-dropout medium and incubated for an additional 3–5 days to assess protein–protein interactions.

### 4.8. Luciferase Assay

The coding sequences of *MsiKNOX09* and *MsiMYB1* were amplified from *M. sieversii* cDNA and inserted into the pCAMBIA1300-nLUC and pCAMBIA1300-cLUC vectors, respectively, to generate fusion expression constructs. These constructs were introduced into *Agrobacterium tumefaciens* and transiently expressed in *Nicotiana benthamiana* leaves via agroinfiltration. For substrate preparation, 25 mg of D-luciferin potassium salt was dissolved in 0.7852 mL of sterile water to obtain a 100 mM stock solution. Prior to use, the stock was diluted with sterile water to a final concentration of 1–5 mM. The diluted substrate was evenly applied to the infiltrated leaf areas. After a 5 min incubation in the dark, luminescence signals were captured using an in vivo plant imaging system (Tanon, Shanghai, China).

## Data Availability

The raw data supporting the conclusions of this article will be made available by the authors on request.

## Supplementary Materials

The following supporting information can be downloaded at https://www.mdpi.com/article/10.3390/plants15081152/s1. Supplementary Figure S1. Multiple sequence alignment of MsiKNOX proteins. Supplementary Figure S2. Sequence logo of the conserved motifs in MsiKNOX proteins. Supplementary Figure S3. Chromosomal distribution of *KNOX* genes in *M. sieversii*. Supplementary Table S1. Information of *MsiKNOX* genes and their encoded proteins identified in the *Malus sieversii* genome. Supplementary Table S2. Genomic information of multiple species. Supplementary Table S3. Primers used in this study.
