# Supplementary material for: Genome-Wide Analysis of the KNOX Gene Family in Malus sieversii
Source: Plants (Basel). 2026 Apr 9;15(8):1152. doi: 10.3390/plants15081152 (PMC13119744; doi:10.3390/plants15081152)
Supplement: Supplementary file 1 [file plants-15-01152-s001.zip › plants-4212000-supplementary.pdf]

Supplementary Figure S1. Multiple sequence alignment of MsiKNOX proteins.

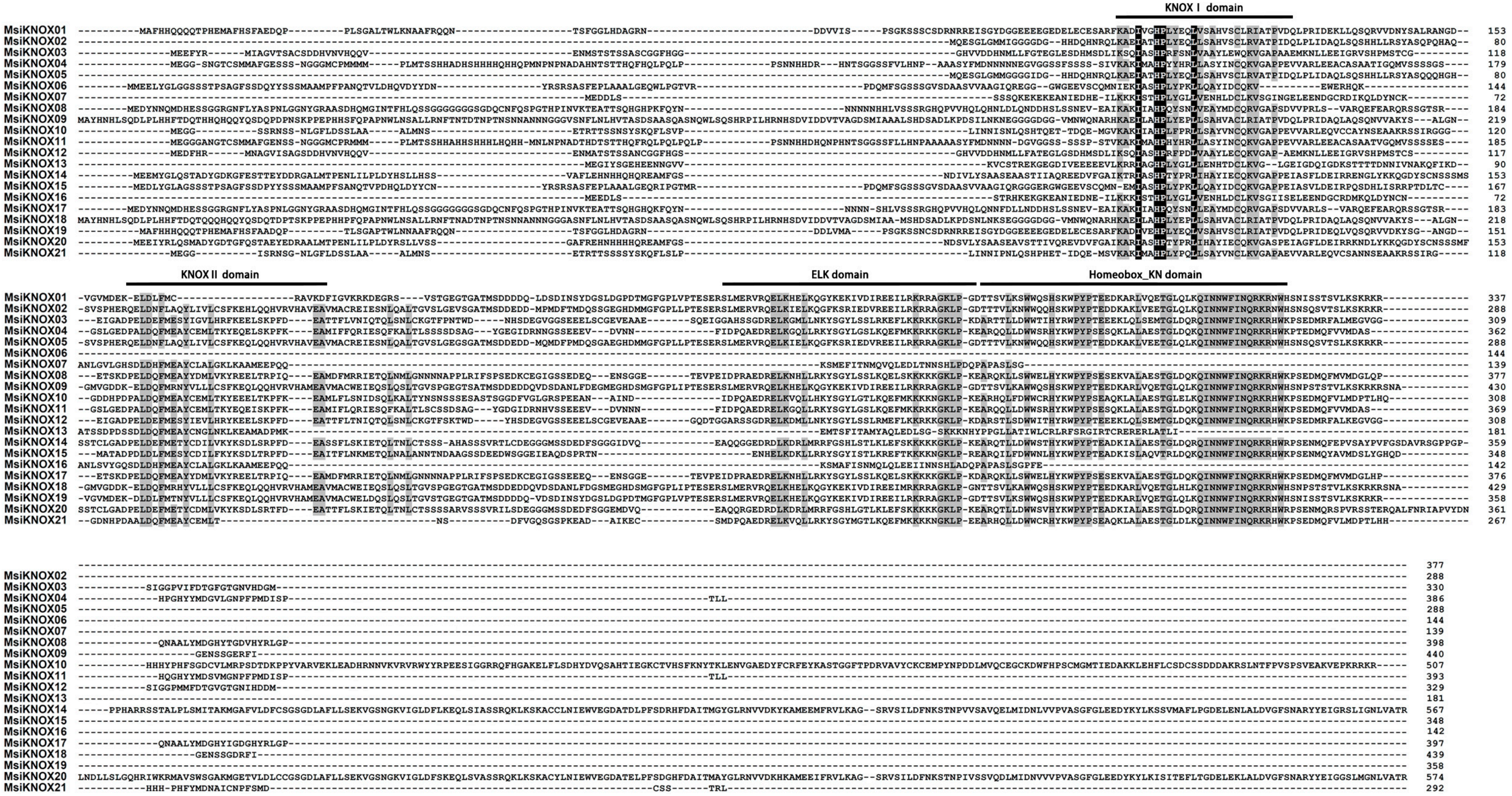

Supplementary Figure S2. Sequence logo of the conserved motifs in MsiKNOX proteins.

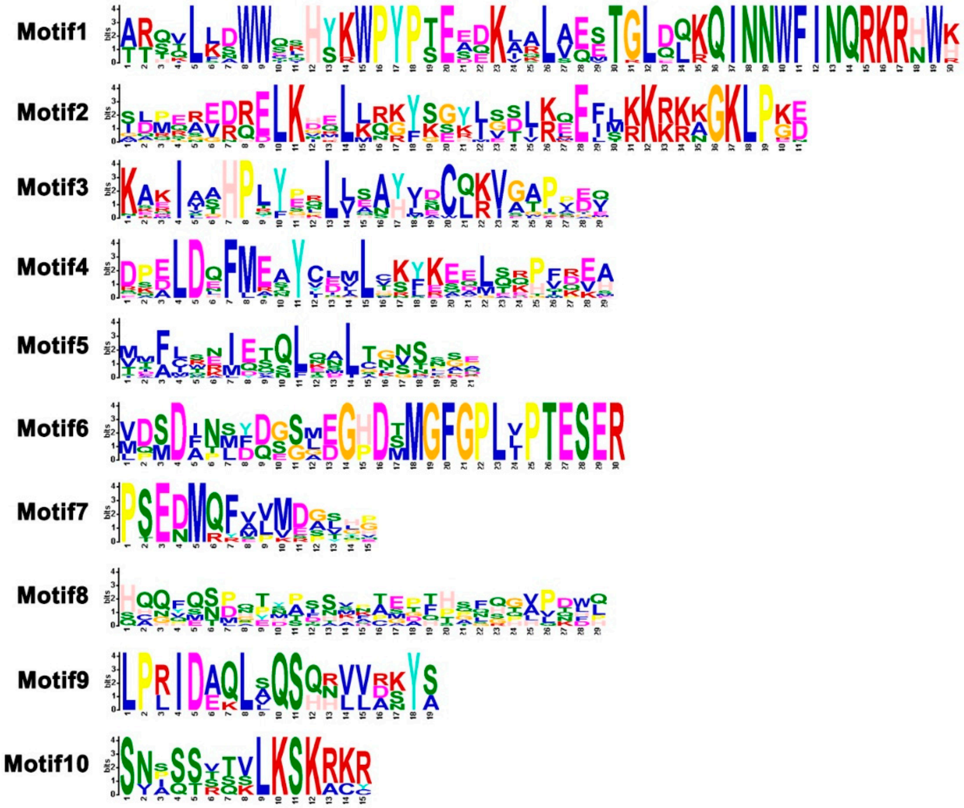

Supplementary Figure S3. Chromosomal distribution of *KNOX* genes in *M. sieversii*

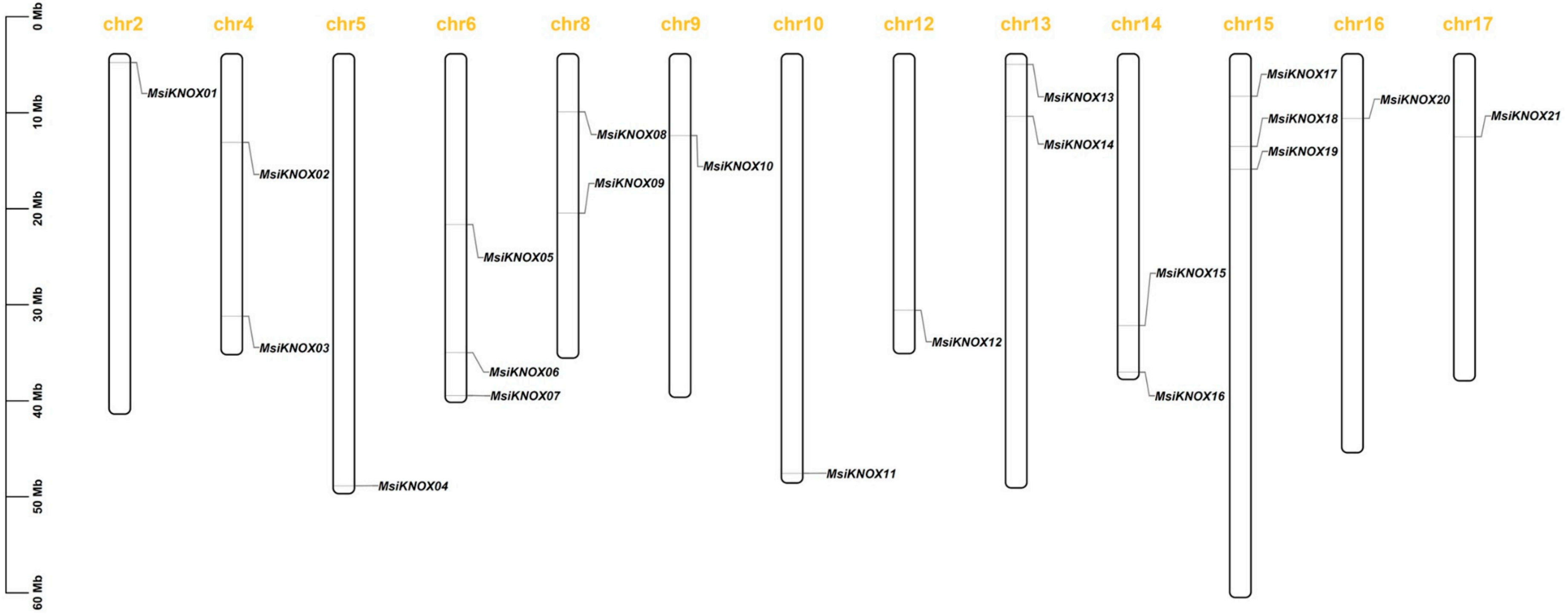

Supplementary Table S1. Information of *MsiKNOX* genes and their encoded proteins identified in the *Malus sieversii* genome.

Gene name	Gene ID	Protein size (aa)	MW (KDa)	pI	GRAVY	Localization prediction
MsiKNOX01	Msi_02g001260	337	38.16	5.66	-0.865	Nucleus
MsiKNOX02	Msi_04g007780	288	32.86	6.31	-0.708	Nucleus
MsiKNOX03	Msi_04g017330	330	36.83	5.14	-0.547	Nucleus
MsiKNOX04	Msi_05g033210	386	43.06	6.25	-0.722	Nucleus
MsiKNOX05	Msi_06g007360	288	32.85	6.31	-0.739	Nucleus
MsiKNOX06	Msi_06g015340	144	15.71	4.67	-0.440	Chloroplast; Nucleus
MsiKNOX07	Msi_06g020260	139	15.51	4.71	-0.633	Cytosol; Nucleus
MsiKNOX08	Msi_08g006890	398	45.16	5.99	-1.017	Cytosol; Nucleus
MsiKNOX09	Msi_08g014360	440	49.28	5.89	-0.786	Cytosol; Nucleus
MsiKNOX10	Msi_09g010460	507	57.74	6.56	-0.713	Nucleus
MsiKNOX11	Msi_10g029830	393	43.91	6.32	-0.734	Nucleus
MsiKNOX12	Msi_12g017880	329	36.83	5.09	-0.645	Nucleus
MsiKNOX13	Msi_13g001460	181	20.26	5.10	-0.556	Cytosol; Nucleus
MsiKNOX14	Msi_13g008320	567	63.08	5.39	-0.404	Nucleus
MsiKNOX15	Msi_14g018170	348	39.31	5.20	-0.692	Nucleus
MsiKNOX16	Msi_14g023230	142	15.94	4.72	-0.543	Cytosol; Nucleus
MsiKNOX17	Msi_15g006310	397	44.99	5.95	-0.984	Cytosol; Nucleus
MsiKNOX18	Msi_15g012660	439	49.09	6.01	-0.799	Cytosol; Nucleus
MsiKNOX19	Msi_15g015210	358	40.55	5.29	-0.769	Nucleus
MsiKNOX20	Msi_16g009000	574	64.61	5.74	-0.437	Nucleus
MsiKNOX21	Msi_17g009530	292	33.10	6.67	-0.614	Nucleus

Supplementary Table S2. Genomic information of multiple species.

Species	Genome ID	Website
M. sieversii	v1.0	https://www.rosaceae.org/
A. thaliana	167	https://phytozome-next.jgi.doe.gov/
M. domestica	491	https://phytozome-next.jgi.doe.gov/
P. betulifolia	None	http://pyrusgdb.sdau.edu.cn/
V. vinifera	457	https://phytozome-next.jgi.doe.gov/
O. sativa	IRGSP-1.0	http://plants.ensembl.org/
S. lycopersicum	514	https://phytozome-next.jgi.doe.gov/

Supplementary Table S3. Primers used in this study.

Name	Sequence
MsiKNOX08-qPCR-f	ACTCTCAGTTGCCAGACAGG
MsiKNOX08-qPCR-r	ACTGATCGAGTTCTGGGTCTT
MsiKNOX09-qPCR-f	ATCAGCGGAAGAGGAACTGG
MsiKNOX09-qPCR-r	TGCGCTTGCTCTTCAAAACC
MsiKNOX12-qPCR-f	TCATGGCAGTGGTCATGTGG
MsiKNOX12-qPCR-r	GATCGGATCCCAAACCCTCC
MsiKNOX13-qPCR-f	CCGACTGTTTGAAGGTGGGT
MsiKNOX13-qPCR-r	GTCTGAAGAATCGGGATCGCT
MsiKNOX19-qPCR-f	GTCTGGGAAGAGCAGCAACT
MsiKNOX19-qPCR-r	GTCTTCCCCCTCCTCCTCTT
MsiKNOX21-qPCR-f	AGGCAGCAAAGAGGAGTAGC
MsiKNOX21-qPCR-r	CCAACAAAATCACTATTGGTCAGC
ACTIN-qPCR-f	ACACGGGGAGGTAGTGACAA
ACTIN-qPCR -r	CCTCCAATGGATCCTCGTTA
MsiMYB1-BD-F	GGAGGACCTGcatatgATGGAGGGATATAACGAAAACC
MsiMYB1-BD-R	GGATCCCCGGgaattcTTCTTCTTTTGAATGATTCCAAAG
MsiKNOX09-AD-F	AGATTACGCTcatatgATGGCGTACCACAACCACCTCTC
MsiKNOX09-AD-R	CACCCGGGTGgaattcCCTTTTGCGCTTGCTCTTCAAA
MsiMYB1-nLUC-F	ACGAGCTCGGTACCCGggatccaATGGAGGGATATAACGAAAACC
MsiMYB1-nLUC-R	CGCGTACGAGATCTGgtcgacTTCTTCTTTTGAATGATTCCAAAG
MsiKNOX09-cLUC-F	GCGGTACCCGggatccaATGGCGTACCACAACCACCTCTC
MsiKNOX09-cLUC-R	AACGAAAGCTctgcagCCTTTTGCGCTTGCTCTTCAAA
